# Dr. Francis L. Shepard

**Published:** 1906-07

**Authors:** 


Dr. Francis L. Shepard, of Buffalo, (U. of B. '96) was found dead on the Erie railway tracks at Batavia, N. Y., May 26, 1906. His age was 39 years. A suspicion of murder was raised, owing to certain mysterious appearances of the case, but later these were cleared up and it became apparent that his death resulted from accident.

